# Role of Qualified Physicians as Antenatal Care Providers in Reducing Birth Complications in Home-delivered Rural Women in Bangladesh

**DOI:** 10.7759/cureus.3974

**Published:** 2019-01-28

**Authors:** Mohammad Abdullah Heel Kafi, Faisal Ahmmed, Md. Zakiul Hassan, Md. Tariqujjaman, Md. Golam Dostogir G Harun

**Affiliations:** 1 Epidemiology and Public Health, International Centre for Diarrhoeal Disease Research, Dhaka, BGD; 2 Internal Medicine, International Centre for Diarrhoeal Disease Research, Dhaka, BGD

**Keywords:** antenatal care, qualified physician, home delivery, rural women, delivery complications, mixed effect, demographic health survey

## Abstract

Introduction

Bangladesh has made significant strides in maternal and neonatal death by ensuring qualified antenatal care (ANC) visits during the pregnancy period of women. To ensure this qualified ANC, the government of Bangladesh has increased the number of qualified physicians and skilled birth attendants at health facilities and encouraged pregnant women to take this eligible ANC during pregnancy. Despite this progress, the majority of deliveries among rural women still occur at home, assisted by traditional birth attendants. These traditional birth attendants at home or even skilled birth attendants at the health facility or home are not always cable of helping women to overcome severe delivery complications. Proper birth preparation before pregnancy through qualified ANC might be a solution here. Taking advice for appropriate birth preparation from a qualified physician (medical doctor) would ensure qualified ANC. In this study, we examined how ANC from a qualified physician, as compared to other trained providers, influences rural women delivering at home to prepare for birth and reduces severe delivery complications.

Methods

The data of 1554 rural women who delivered at home were extracted from the 2014 Bangladesh Demographic and Health Survey data. A mixed-effects logistic regression model was carried out for the binary delivery complications data, to assess the influence of qualified physicians as ANC providers on delivery complications by adjusting the effect of other socio-demographic covariates and clustering.

Results

Of the women from rural areas who delivered at home, 42% reported delivery complications. Those who received ANC from a qualified physician were 32% less likely (OR 0.68; 95% CI 0.50, 0.91) to report facing delivery complications as compared to those who had received ANC from other trained or unqualified providers adjusted by socio-demographic determinants in Bangladesh.

Conclusions

Developing a sustained and effective strategy could be a precedent for promoting ANC from qualified physicians for rural women delivering at home, to decrease delivery complications as well as creating healthy environments for safe deliveries.

## Introduction

Globally, maternal mortality due to delivery complications remains unacceptably high, especially among women in low-income countries [1]. Every day, approximately 830 women die from pregnancy and childbirth-related causes and almost 99% of all maternal deaths occur in low-resource settings [2]. Moreover, complications as a result of the lack of suitable delivery management result in about 10-20 million women worldwide developing physical or mental disabilities each year [3]. Over half of deliveries occur at home in Bangladesh [4]. Although Bangladesh's maternal and infant mortality has declined due to better health care planning throughout the country [5], approximately 5000 women continue to die every year in Bangladesh due to pregnancy or birth-related causes [6]. Most of these delivery-related deaths occur in rural households under the care of unqualified birth attendants [4].

Despite the Government of Bangladesh’s initiative to increase the number of qualified physicians and midwives in health facilities [7] in both rural and urban areas, rural women are still reluctant to go to health facilities [4] and continue to deliver at home with traditional birth attendants. Although birth attendants can sometimes aid in emergency at-home deliveries, some complications cannot be dealt with without access to health facilities [8]. This causes women living in rural areas to face complications, as they do not have enough knowledge regarding birth preparation. In this regard, in addition to the presence of skilled birth attendants, the woman or her family member also need adequate knowledge on possible complications related to delivery, and this is only achievable through healthier antenatal care visits (ANC) for the duration of the pregnancy [9].

ANC is a clinical process of care provided by health care providers to women during their pregnancy to educate them about various danger signs and symptoms, which can significantly improve their own health and that of their infants during pregnancy, delivery, and in the postpartum period [10]. The World Health Organization (WHO) recommends a minimum of four ANC visits but, regrettably, only about 60% of all pregnant women worldwide follow this recommendation. In sub-Saharan Africa and South Asia, where maternal mortality is the highest, about 52% of women in sub-Saharan Africa and 46% of women in South Asia received at least four antenatal visits [11-12]. Recently, the WHO replaced the four visits ANC-focused model and suggested a minimum of eight visits to a healthcare provider throughout the pregnancy [13]. In 2014, 78% of women in Bangladesh received at least one ANC session from any provider, whereas in 2004, 2007, and 2011, it was 58%, 63%, and 68%, showing substantial improvement [4,14-16].

In Bangladesh, ANC providers can be qualified and unqualified based on their academic qualifications. Qualified physicians who received a medical degree approved by the Government of Bangladesh are considered qualified providers. Nurses, midwives, and community health care workers are regarded as trained providers whereas unqualified physicians and NGO workers are considered unqualified ANC providers [4]. In Bangladesh, midwives and medically trained providers are considered qualified providers, but their role in increasing the number of ANC visits and taking birth preparation was small ( only 4% percentage, from 51% in 2011 to 55% in 2014) [4]. Similar to other low and middle-income countries, many women in Bangladesh die each year because of the lack of proper pregnancy and delivery preparedness. This happens mostly in rural areas [4,17-18].

Compared to other providers, qualified ANC providers are better equipped to monitor the status of pregnancy, identify complications associated with pregnancy, and give advice on the steps of birth preparation to prevent adverse pregnancy outcomes [19-21]. In Bangladesh, many studies explored the beneficial effects of receiving the required number of ANC visits from midwives and having skilled birth attendants during deliveries at health facilities as well as at home to reduce delivery complications. However, no extensive research has yet been done to explore the advantageous health effects (if any) of receiving ANC from qualified physicians, especially in the rural areas of Bangladesh. This study, based on nationally representative data, aimed to investigate this while adjusting for other risk factors. Findings from this exercise might be helpful for family planning professionals to take better steps to mitigate maternal and infant mortality due to delivery complications.

## Materials and methods

Study design

The data set for this study was extracted from the Bangladesh Demographic and Health Survey (BDHS) of 2014, a nationally representative cross-sectional survey conducted by the National Institute of Population Research and Training (NIPORT), ICF International (USA), and Mitra and Associates between June and November 2014 [4].

Sampling technique

The sampling method was a two-stage stratified sample of households. Firstly, 600 enumerations areas (EAs) were selected with probability proportional to size at the first stage, with 207 urban and 393 rural EAs. An EA is defined as a village, a group of small villages, or part of a large village. Second, a systematic sample of 30 households on average was selected per EA to provide statistically reliable estimates of the key demographic and health variables [4]. The 2014 BDHS reported information on 17,863 households through questionnaires with women. Using this, we separated the information of 12,816 women from rural areas and selected 11,666 who had at least one child during the survey. From this group, we extracted information about the place of delivery from 2548 women and finally selected 1554 women who had home births. Written informed consent was obtained from the participants prior to participation in the study and data collection was conducted confidentially. For this study, there was no need for ethical approval since it is based on publicly available secondary data.

Variable assessed and measured

Our targeted population was rural women who delivered at home. They were asked about their demographic information (age, sex, education level, household head education level, division (administrative area), wealth status) and information on ANC providers. In this study, we categorized the ANC providers into three groups: Qualified physicians, who are actually medical doctors, obtained a Bachelor of Medicine and Bachelor of Surgery (MBBS) degree from Bangladesh government-approved medical colleges, other trained providers who was nurses, midwives, paramedics, family welfare visitors, community skilled birth attendants, medical assistants of medical officers, and unqualified providers who worked as community health care providers, untrained traditional birth attendants, non-government organization (NGO) workers, and family welfare assistants, as they were reported in the 2014 BDHS report [4]. We furthermore investigated information on the health-care-seeking behavior of women during their pregnancy such as iron supplements, weight, blood, and urine samples were taken and education on signs of danger during delivery.

Outcome variable measure

Complications during delivery are generally any problems that pose a risk to the health of the mother and/or baby [4]. In the 2014 BDHS report, there were no details of the various complications; rather, women were asked whether they faced any complications or not during their last birth, preceding the survey. We used this reported information from study participants as our outcome variable. Here, the complications of women included labor pain, the possibility of a premature baby, bleeding, unsafe termination, and pre-eclampsia. Since analyses using the 2014 BDHS data require that sampling weights be applied to ensure the applicability of the survey results at the national and domain levels, we considered the weight variable while constructing the outcome variable. A brief calculation and explanation of the weight variable can be found in the BDHS 2014 report [4].

Statistical analysis

We analyzed the socio-demographic characteristics of targeted women, such as age, number of children, age at first birth, education, education of the household head, wealth index, division, and occupation, using descriptive statistics. We also explored the health care behavior of the women during their pregnancy, whether they took iron or folic acid supplements, the number of ANC visits, types of ANC providers, whether their last child was wanted at the time, their height, body mass index (BMI; details of this calculation are described elsewhere) [4] and if they faced complications during their last pregnancy. With the outcome variable of whether or not women faced complications at delivery, we calculated the odds ratio using mixed-effect logistic regression to measure the association between the type of ANC provider during pregnancy and reported complications faced at the time of home delivery. We used a mixed-effect logistic regression analysis because, in the BDHS 2014 report, data is nested in nature.

Women's different socio-demographic characteristics were depicted in a conceptual framework to present possible confounders and to find the adjusted effect of ANC providers on complications faced by women. We performed multivariable logistic regression, where the adjustment was done by allowing for these confounder variables. All the analysis was performed using R statistical software (version 3.3.1) and Stata software (Stata/SE 13.1, College Station, TX 77845, USA).

## Results

Demographic characteristics of women who had home deliveries (n=1554)

Among 1554 rural women who delivered at home, about 42% reported facing complications at the time of their last delivery. Most of these home-delivered rural women (60%) were aged between 20 and 30 years whereas 16% were over 30 years old. The prevalence of home delivery was highest in Dhaka (34%) and Chittagong (19%) divisions. The majority of these women (52%) had completed their secondary education and 33% of women had a partner who had accomplished primary education only. Women from the poorest (according to the wealth index) family accounted for a higher percentage (26%) of home deliveries whereas the richest accounted for only 7% (Table 1).

**Table 1:** Demographic and socio-economic information of home delivered rural women and their delivery complication

Demographic Characteristics	Women who had taken home delivery	Faced complication during delivery at last birth	
	n (%)	Yes, n (%)	No, n (%)
	1554 (61)	653 (42)	901(58)
Age of the respondents			
15-19	369(24)	148 (23)	221 (25)
20-24	491(32)	199 (30)	292 (32)
25-30	442(28)	197 (30)	245 (27)
Above 30	252(16)	109 (17)	145 (16)
Division			
Dhaka	533(34)	215 (32)	318 (35)
Chittagong	303(19)	131(20)	171 (19)
Barisal	102(7)	51 (8)	51 (6)
Khulna	116(8)	49(8)	67 (7)
Sylhet	148(9)	57 (9)	91 (10)
Rajshahi	155(10)	58 (9)	97 (11)
Rangpur	197(13)	91 (14)	106 (12)
Education of the respondents			
No-education	224 (15)	82 (13)	142 (16)
Primary	465 (30)	215 (33)	250 (28)
Secondary	799 (51)	327 (50)	472 (52)
Higher	66 (4)	30 (5)	37 (4)
Education of the husband/ household head			
No-education	451 (29)	211 (32)	240 (27)
Primary	516 (33)	205 (32)	311 (35)
Secondary	473 (30)	191 (29)	282 (31)
Higher	114 (8)	46 (7)	68 (8)
Wealth index			
Poorest	401 (26)	161 (25)	240 (27)
Poorer	392 (25)	176 (27)	216 (24)
Middle	380 (24)	153 (23)	227 (25)
Richer	283 (18)	112 (17)	171 (19)
Richest	98 (7)	51 (8)	47 (5)

Demographic characteristics of rural women who had home deliveries and faced delivery complications (n=653)

**Figure 1:**
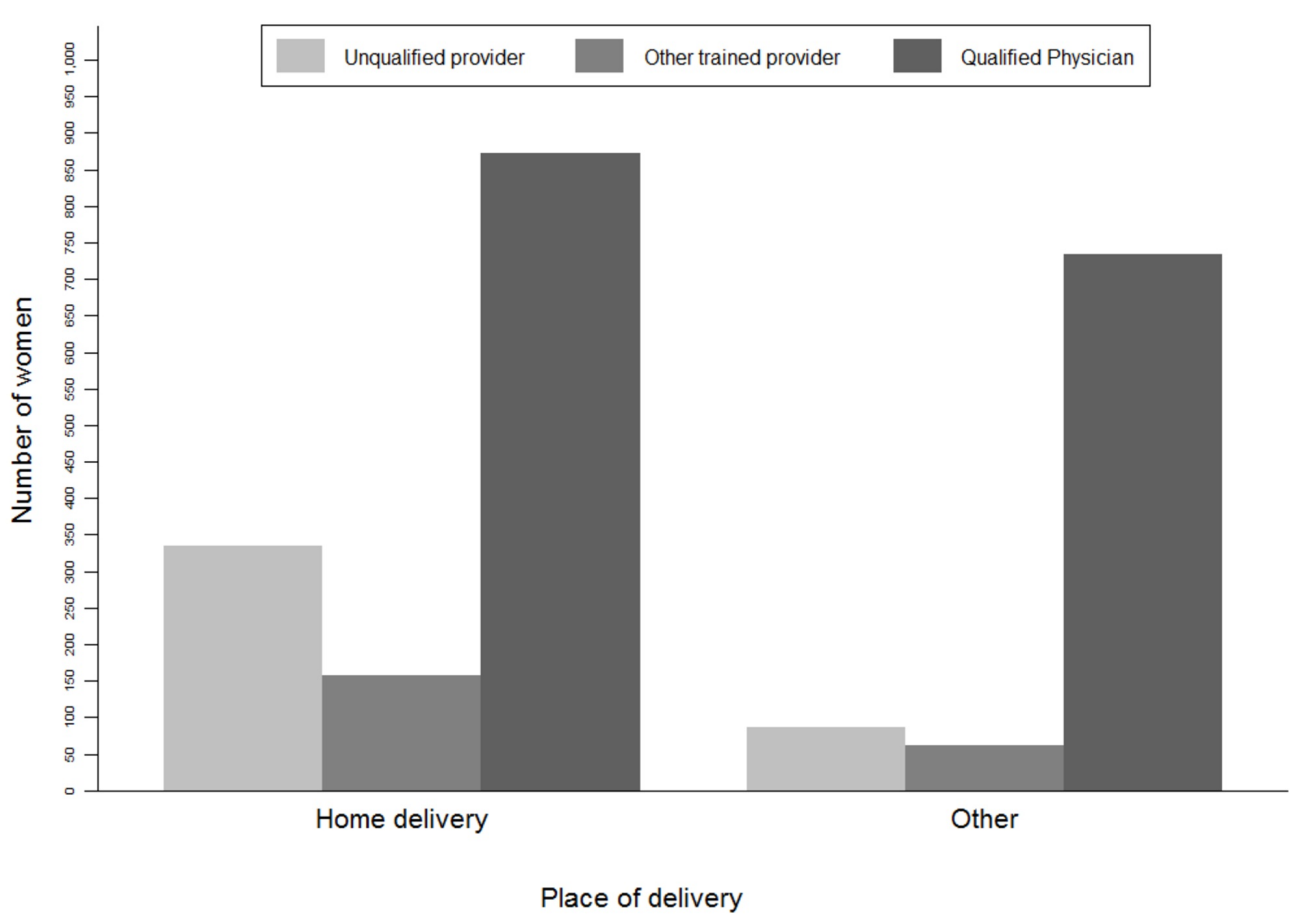
Distribution of ANC provider based on delivery place of the study women ANC: antenatal care

**Figure 2:**
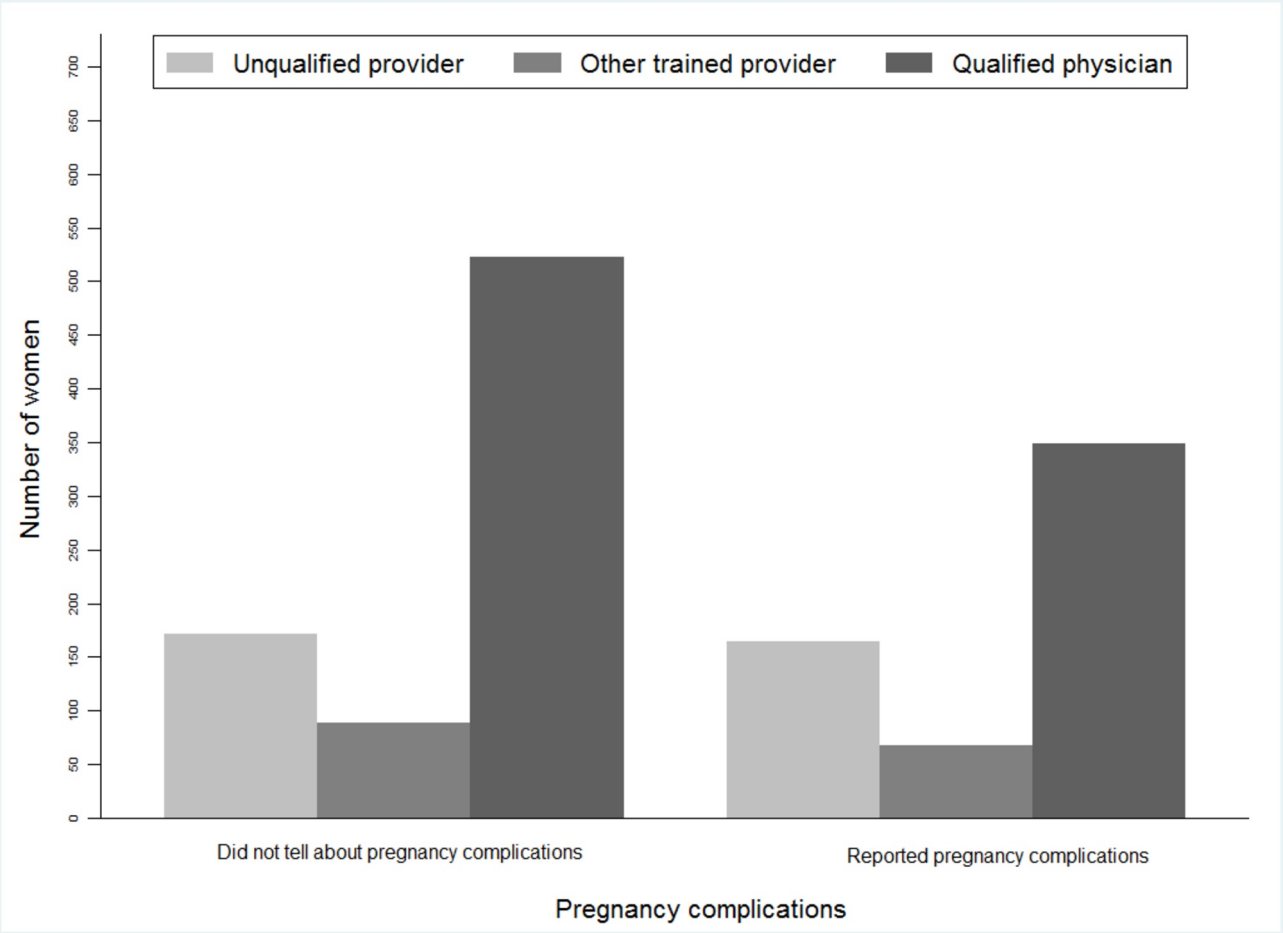
Distribution of ANC provider based on reported information regarding delivery complications of the study women ANC: antenatal care

Women who reported facing delivery complications, 61% of them were between 20 and 30 years old. The prevalence of women who faced complications was the highest (33%) in Dhaka. This may be due to the fact that awareness of reporting of complications was more prevalent in Dhaka, enabling them to take preventive measures for birth preparation before delivery. On the other hand, most of the women (83%) who completed their primary and secondary education faced complications. We found similar distributional patterns of delivery complications among those women (61%) whose husbands had completed primary and secondary education. We also noted that the prevalence of delivery complications varied across the wealth status of the respondents. Delivery complications were mostly (25%) reported by women from poorer families whereas only 8% of the richest women reported complications (Table 1). Most of the respondents in both categories (home and facility deliveries) had received ANC from qualified physicians during their pregnancy (Figure 1). Women who had received ANC from qualified physicians reported fewer delivery complications than those who consulted unqualified health care providers (Figure 2).

Characteristics during pregnancy of rural women who had home deliveries (n=1554)

Table 2 provides an indication of antenatal history as well as other birth preparations of rural women who delivered at home. The majority of women (93%) did not take iron supplements during pregnancy. More than half of the women (70%) had less than four ANC visits, which is lower than previous WHO recommendations of at least four visits (WHO recently changed this number to eight visits) [13]. About 84% of the women were shorter than 145 cm. Over half of the women (61%) had normal weight while 27% were underweight based on the body mass indexed value. Sixty-three percent of the women received ANC from qualified physicians whereas 27% received ANC from unqualified ANC providers. About 52% of the women were well-informed on the signs of danger during delivery while 57% had undergone ultrasound tests during the pregnancy.

**Table 2:** Characteristics of study women during pregnancy

Characteristics	Women who had taken home delivery	Faced complication during delivery at last birth	
	n(%)	n(%)	
	1554 (61)	Yes, n (%)	No, n (%)
		653 (42)	901(58)
Took iron tablet			
Yes	68 (5)	34(5)	34(4)
No	1443(93)	597(93)	846(96)
ANC attendance			
< 4 times	1093(70)	404(62)	689(77)
≥ 4 times	460(30)	249(38)	211(23)
ANC provider			
Unqualified	422(27)	210(32)	212(24)
Other trained provider	160(10)	64(10)	97(11)
Qualified doctor	972 (63)	380(58)	592(66)
Last child intended			
Wanted then	1112 (72)	497 (76)	615(68)
Wanted later	233(15)	74(11)	159(18)
Wanted no more	207(13)	82(12)	126(13)
Height (% who's height is less than 145cm)			
Yes	224(14)	105(16)	120(13)
No	1319(85)	544(84)	775(87)
Body mass index (BMI)			
Underweight	419(27)	163(25)	256(29)
Normal weight	944(61)	406(63)	539(60)
Overweight	179(12)	80(12)	99(11)
Counsel about danger signs of delivery			
Yes	805(52)	544 (83)	261 (29)
No	749(48)	109(17)	640(71)
Take ultrasound test			
Yes	885(57)	348(53)	537(60)
No	669(43)	305(47)	364(40)

Characteristics during pregnancy of rural women who had home deliveries and faced delivery complications (n=653)

Among the women who faced complications, the majority (93%) of them did not take any iron supplements during their last pregnancy. A lower percentage of women (38%) who had at least four ANC visits reported facing delivery complications. A higher number of women (50% (210 out of 422 women)) who had received ANC from unqualified providers reported facing delivery complications whereas only 39% (380 out of 972 women) who had gone to qualified physicians had delivery complications. About 76% of the women desired the last birth willingly. A higher percentage of women (84%) who were less than 145 cm in height reported complications compared to those taller than this height range. Among those who were counseled about the signs of danger during delivery, 83% reported delivery complications while 53% of women counseled on delivery risks underwent ultrasound tests.

Factors associated with delivery complications in rural women who delivered at home (n=653)

The factors associated with delivery complications in rural women in home births during their last birth are presented in Table 3. The main factor that we wanted to explore in this study was the effect of qualified physicians’ advice on reducing delivery hurdles during ANC visits as compared to advice taken from other types of health care providers. We observed that during ANC visits, women who took advice from qualified physicians were 32% (OR 0.68; 95% CI 0.50, 0.91) less likely to face delivery complications as compared to those who had taken advice from unqualified providers adjusted by other confounder variables. This is because as compared to unqualified ANC providers, women who took advice from the qualified physicians got better guidance regarding the symptoms of complications and, therefore, they took better delivery precautions.

**Table 3:** Factors associated with complications faced by rural women delivered at home at the time of their last birth within the three years preceding the survey, 2014 (n = 653)

Characteristics	Crude OR(95% CI)	P - value	Adjusted OR(95% CI)	P-value
Age of the respondents				
15-19	1			
20-24	1.05(0.76-1.46)	0.739		
25-30	1.19(0.85-1.67)	0.294		
Above 30	1.18(0.80-1.74)	0.397		
Division				
Barisal	1			
Chittagong	0.74(0.45 - 1.23)	0.252		
Dhaka	0.71 (0.43 - 1.20)	0.206		
Khulna	0.65 (0.37 - 1.14)	0.139		
Sylhet	0.48 (0.28 - 0.82)	0.008		
Rajshahi	0.54 (0.31 - 0.95)	0.033		
Rangpur	0.81 (0.48 - 1.37)	0.446		
Education of the respondent				
No-education	1		1	
Primary	1.31(0.88 - 1.95)	0.180	1.50(0.99-2.28)	0.055
Secondary	1.33(0.91 - 1.95)	0.134	1.66(1.06-2.58)	0.025
Higher	1.43(0.76 - 2.62)	0.27	1.95(0.95-4.03)	0.071
Education of the husband/ household head				
No-education	1		1	
Primary	1.08 (0.80 - 1.47)	0.611	1.04(0.76-1.42)	0.783
Secondary	0.93 (0.68 - 1.29)	0.702	0.86(0.62-1.20)	0.393
Higher	1.09 (0.74 - 1.63)	0.375	0.72(0.43-1.18)	0.192
Wealth index				
Poorest	1		1	
Poorer	1.22 (0.88 - 1.69)	0.227	1.19(0.85-1.66)	0.314
Middle	1.23 (0.87 - 1.73)	0.239	1.18(0.81-1.70)	0.375
Richer	1.10 (0.75 -1.61)	0.593	1.09(0.72-1.65)	0.664
Richest	1.40 (0.82 -2.40)	0.223	1.42(0.79-2.54)	0.232
Took iron tablet				
No	1		1	
Yes	1.43(0.80-2.60)	0.229	1.49(0.73-3.08)	0.272
ANC attendance				
< 4 times	1		1	
≥ 4 times	2.21 (1.67 - 2.89)	0.001	2.10(1.58-2.78)	0.001
ANC provider				
Unqualified	1		1	
Other trained provider	0.87 (0.56 - 1.34)	0.530	0.88(0.57 - 1.37)	0.592
Qualified physician	0.69 (0.52 - 0.92)	0.014	0.68(0.50 - 0.91)	0.012
Last child intended				
Wanted no more	1		1	
Wanted then	1.40(0.96 - 2.03)	0.075	1.54 (1.02 - 2.33)	0.039
Wanted later	1.02(0.64- 1.63)	0.916	1.16 (0.70 - 1.93)	0.565
Height (% who's height is less than 145cm)				
No	1		1	
Yes	1.05(0.74-1.50)	0.776	1.06(0.74-1.51)	0.744
Body mass index (BMI)				
Normal weight	1		1	
Underweight	0.96(0.74-1.27)	0.821	0.98(0.74-1.28)	0.892
Overweight	1.20(0.80-1.79)	0.392	1.16(0.77-1.75)	0.456
Counsel about danger signs of delivery				
No	1		1	
Yes	17.09(12.31 - 23.72)	0.001	16.33(11.75 - 22.71)	0.001
Took ultrasound test				
No	1		1	
Yes	0.91(0.72 - 1.16)	0.449	1.10 (0.78 - 1.56)	0.568

We also observed that as compared to women aged 15 to 19 years old, the risk of facing delivery complications in women aged 20 to 24 years, 25 to 30 years, and over 30 years were 1.05 (95% CI 0.76,1.46), 1.19 (95% CI 0.85,1.67), and 1.18 (95% CI 0.80,1.74) times higher. Higher educated women were more likely (OR 1.95; 95% CI 0.95, 4.03) to report facing delivery complications as compared to women without education adjusted by age, living place, division, husband's education, and wealth status of the family. This may be due to their awareness and willingness to find a solution to complications. Similar awareness was found among those women (OR 1.04; 95% CI 0.76, 1.42) whose husbands had at least completed primary education as compared to those whose partner was not educated after adjustment done by the wealth status of the family. Analogously, women with a higher wealth status due to responsiveness and enthusiasm to overcome delivery obstacles were 42% (OR 1.42; 95% CI 0.79, 2.54) more likely to report complications than women from poorer families during the ANC period.

Women who faced more complications during pregnancy were prone (OR 2.10; 95% CI 1.58, 2.78) to look for further ANC than the recommended number of ANC visits (at least four) from health care providers, to find solutions for their complications, which may be due to their consciousness and awareness. A similar willingness to report delivery complications (OR 1.49; 95% CI 0.73, 3.08) was found in those women who took iron supplements during pregnancy. Body mass index (BMI) is also a good indicator of women’s health. In our study, we observed that women who were overweight (based on the BMI scale) as compared to normal weight reported higher (OR 1.16; 95% CI 0.77, 1.75) whereas those who were underweight reported less (OR 0.98; 95% CI 0.74, 1.28) about their delivery complications. We also found that due to alertness, women who underwent ultrasound tests and were counseled about the signs of danger during delivery were more likely (OR 16.33 95% CI 11.75, 22.71 and OR 1.10 95% CI 0.78, 1.56) to report delivery complications as compared to those who did not receive the ultrasound test and counseling.

## Discussion

Childbirth in low resource settings, such as rural areas in Bangladesh, especially in the case of home delivery without the presence of a qualified health provider [4], can prove dangerous and increase the chances of delivery complications [22]. Fortunately, the Government of Bangladesh, which was inspired by the earlier success of adding more midwives in health facilities, has again employed more midwives and qualified physicians to overcome the challenges of reducing maternal as well as infant death as part of the Millennium Development Goals [23].

Despite the government’s efforts, the availability of qualified birth attendants in rural homes remains low [23] and the lack of sufficient birth preparedness continues to lead to delivery complications [24]. In this situation, women have to prepare from the very beginning of her pregnancy. Hence, more focus on receiving improved ANC visits is needed so that rural women would be able to receive counseling on birth preparedness and make necessary arrangements during home births. Improved ANC would be possible when women receive counseling from qualified physicians at a health facility [25]. Considering this, our study highlighted how the type of health care provider during ANC can play an imperative role in reducing delivery complications based on the nationally representative demographic survey in 2014.

We found, after analyzing the data, that rural women who had home births during their last birth and received ANC from qualified physicians were 32% less likely to report delivery complications in comparison to those who consulted unqualified providers. In this case, earlier notification about complications would be possible through ANC from qualified physicians. Similar findings in other settings [26] reported that qualified health care providers during ANC visits led to women experiencing safe deliveries at home. Besides the type of ANC providers, we observed that, like other studies, high ANC attendance [27] led to fewer complications in contrast with those that did not fulfill the required number of ANC visits. Similarly, fewer women who took iron tablets, as advised by qualified physicians during ANC visits, reported fewer delivery complications as other studies reported [28].

Moreover, in our study, when we explored of the pattern of receiving ANC from qualified physicians, we revealed that among all women, only 5% with higher education reported delivery complications, implying that education may promote receiving ANC from qualified physicians. Therefore, the educational development of women as well as of their husbands is necessary to promote healthier behaviors during pregnancy, to overcome birth complications. More women (45%) from poor families reported facing delivery complications. Similar findings also suggested that women died during delivery mostly if they were from a poorer family and a rural area. In this regard, besides engaging more skilled birth attendants at rural health facilities, the government of Bangladesh should also focus on raising awareness among rural women about pregnancy and delivery complications, provide free access to health facilities with qualified physicians to take birth preparation, and conduct monthly door-to-door health services. Then, delivery complications among rural home-delivered women might be reduced.

In our study, we used rigorous statistical techniques by considering individual sampling weights and capturing the clustered dependency among them due to the nesting data structure. We applied the mixed-effect logistic regression modeling technique to correctly identify the factors associated with how pregnant women could minimize delivery complications through receiving ANC from qualified physicians, even for home births, which has given our study more strength as compared to other, similar studies. While other biological and social factors are also relevant, this is the first study where we have revealed that receiving ANC from qualified physicians significantly helps women living in rural areas to experience fewer complications and have safe births.

The main limitation of this study was the lack of information regarding the different types of delivery complications women faced during home births. Other limitations include the cross-sectional nature of the data and small sample size, which made determining the causative relationships between ANC providers and delivery complications in home-births difficult. A further study is needed to capture the various biological and socio-economic factors and hurdles women face during home births so the government can take the appropriate and necessary steps to overcome this health problem in Bangladesh.

## Conclusions

Home-birth complications faced by women in low resource settings, such as rural areas in Bangladesh, are a major public health concern and one of the foremost barriers to achieving the sustainable developmental goal of reducing maternal and infant mortality. The Government of Bangladesh has planned to address this problem by raising awareness and employing more midwives and qualified physicians in rural health facilities. However, these initiatives are facility-based improvements whereas the majority of deliveries continue to occur at home in rural areas, with untrained or unqualified birth attendants. Therefore, improving the availability of quality ANC with qualified physicians in health facilities and encouraging rural women to take ANC visits from a qualified physician during the pregnancy period, to take proper birth preparation, would mitigate the delivery complications and ensure safer births. We anticipate that this outcome will help policymakers and researchers to incorporate and highlight the importance of taking advice from qualified physicians during the pregnancy period in their existing family planning program, to ensure safe births.
